# Supplementary material for: Acid-base and metabolic parameters and cerebral oxygenation during the immediate transition after birth—A two-center observational study
Source: PLoS One. 2023 May 17;18(5):e0283278. doi: 10.1371/journal.pone.0283278 (PMC10191326; doi:10.1371/journal.pone.0283278)
Supplement: Attachment — Submitted filename: PONE-D-22-00810 comments.pdf [file pone.0283278.s002.pdf]

PLOS ONE

Acid-base and metabolic parameters and cerebral oxygenation during the immediate transition after birth - An two-center observational study

--Manuscript Draft--

**Manuscript Number:**	PONE-D-22-00810
**Article Type:**	Research Article
**Full Title:**	Acid-base and metabolic parameters and cerebral oxygenation during the immediate transition after birth - An two-center observational study
**Short Title:**	Acid-base status and metabolism and cerebral oxygenation after birth.
**Corresponding Author:**	Gerhard Pichler<br>Medical University of Graz: Medizinische Universitat Graz<br>Graz, AUSTRIA
**Keywords:**	Acid-base and metabolic parameters; cerebral oxygenation; preterm and term neonates
**Abstract:**	Objective<br><br>The association between blood glucose level and cerebral oxygenation (cerebral regional oxygen saturation [crSO 2] and cerebral fractional tissue oxygen extraction [FTOE]) in neonates has already been described. Aim of the present study was to investigate if acid-base and other metabolic parameters have an impact on cerebral oxygenation immediately after birth in preterm and term neonates.<br><br>Study design<br><br>Post-hoc analyses of secondary outcome parameters of two prospective observational studies were performed. Preterm and term neonates born by caesarean section were included, in whom i) cerebral near-infrared spectroscopy (NIRS) measurements were performed during the first 15 minutes after birth and ii) a capillary blood gas analysis was performed between 10 and 20 minutes after birth. Vital signs were routinely monitored with pulse oximetry (arterial oxygen saturation [SpO 2] and heart rate [HR]). Correlation analyses were performed to investigate potential associations between acid-base and metabolic parameters (lactate [LAC], pH-value [pH], base-excess [BE] and bicarbonate [HCO 3]) from capillary blood and NIRS-derived crSO 2 and FTOE at 15 minutes after birth.<br><br>Results<br><br>One-hundred-fifty-seven neonates, 42 preterm neonates (gestational age [IQR] 34.0 weeks [3.3], birth weight [IQR] 1845g [592]) and 115 term neonates (gestational age [IQR] 38.9 weeks [1.0], birth weight [IQR] 3230g [570]) were included in the study.<br><br>Median crSO 2 [IQR] at 15 minutes after birth were 82% [16] in preterm neonates and 83% [12] in term neonates. Median FTOE [IQR] values at 15 minutes after birth were 0.13 [0.15] in preterm neonates and 0.14 [0.14] in term neonates.<br><br>In preterm neonates, higher LAC and lower pH, and BE were associated with lower crSO 2 and higher FTOE. In term neonates, higher HCO 3 was associated with higher FTOE.<br><br>Conclusion<br><br>There were significant associations between several acid-base and metabolic parameters and cerebral oxygenation in preterm neonates, while in term neonates only HCO 3 correlated positively with FTOE.
**Order of Authors:**	Christian Mattersberger
	Nariae Baik-Schneditz

Powered by Editorial Manager® and ProduXion Manager® from Aries Systems Corporation

	Bernhard Schwaberger
	Georg M. Schmölzer
	Lukas Mileder
	Berndt Urlesberger
	Gerhard Pichler
**Additional Information:**	
**Question**	**Response**
**Financial Disclosure**<br><br>Enter a financial disclosure statement that describes the sources of funding for the work included in this submission. Review the submission guidelines for detailed requirements. View published research articles from *PLOS ONE* for specific examples.<br><br>This statement is required for submission and **will appear in the published article** if the submission is accepted. Please make sure it is accurate.<br><br>**Unfunded studies**<br>Enter: *The author(s) received no specific funding for this work.*<br><br>**Funded studies**<br>Enter a statement with the following details:<br>• Initials of the authors who received each award<br>• Grant numbers awarded to each author<br>• The full name of each funder<br>• URL of each funder website<br>• Did the sponsors or funders play any role in the study design, data collection and analysis, decision to publish, or preparation of the manuscript?<br>• **NO** - Include this sentence at the end of your statement: *The funders had no role in study design, data collection and analysis, decision to publish, or preparation of the manuscript.*<br>• **YES** - Specify the role(s) played.<br><br>* typeset	GP is a recipient of the the Culture Department of the City of Graz, Austria.<br>GMS is a recipient of the Heart and Stroke Foundation/University of Alberta Professorship of Neonatal Resuscitation, a National New Investigator of the Heart and Stroke Foundation Canada and an Alberta New Investigator of the Heart and Stroke Foundation Alberta. This research has been facilitated by the Women and Children's Health Research Institute through the generous support of the Stollery Children's Hospital Foundation.
**Competing Interests**<br><br>Use the instructions below to enter a competing interest statement for this submission. On behalf of all authors,	The authors have declared that no competing interests exist.

disclose any competing interests that could be perceived to bias this work—acknowledging all financial support and any other relevant financial or non-financial competing interests.<br><br>This statement is required for submission and **will appear in the published article** if the submission is accepted. Please make sure it is accurate and that any funding sources listed in your Funding Information later in the submission form are also declared in your Financial Disclosure statement.<br><br>View published research articles from *PLOS ONE* for specific examples.<br><br>**NO authors have competing interests**<br><br>Enter: *The authors have declared that no competing interests exist.*<br><br>**Authors with competing interests**<br><br>Enter competing interest details beginning with this statement:<br><br>*I have read the journal's policy and the authors of this manuscript have the following competing interests: [insert competing interests here]*<br><br>* typeset	
**Ethics Statement**<br><br>Enter an ethics statement for this submission. This statement is required if the study involved:<br><br>• Human participants<br>• Human specimens or tissue<br>• Vertebrate animals or cephalopods<br>• Vertebrate embryos or tissues<br>• Field research<br><br>Write "N/A" if the submission does not require an ethics statement.	This study was approved by the Ethics Committee of Graz, Austria (27-465 ex 14/15) and the Ethics Committee of Edmonton, Canada (Pro00032233)

Powered by Editorial Manager® and ProduXion Manager® from Aries Systems Corporation

General guidance is provided below. Consult the submission guidelines for detailed instructions. **Make sure that all information entered here is included in the Methods section of the manuscript.**<br><br>**Format for specific study types**<br><br>**Human Subject Research (involving human participants and/or tissue)**<br>• Give the name of the institutional review board or ethics committee that approved the study<br>• Include the approval number and/or a statement indicating approval of this research<br>• Indicate the form of consent obtained (written/oral) or the reason that consent was not obtained (e.g. the data were analyzed anonymously)<br><br>**Animal Research (involving vertebrate animals, embryos or tissues)**<br>• Provide the name of the Institutional Animal Care and Use Committee (IACUC) or other relevant ethics board that reviewed the study protocol, and indicate whether they approved this research or granted a formal waiver of ethical approval<br>• Include an approval number if one was obtained<br>• If the study involved *non-human primates*, add *additional details* about animal welfare and steps taken to ameliorate suffering<br>• If anesthesia, euthanasia, or any kind of animal sacrifice is part of the study, include briefly which substances and/or methods were applied<br><br>**Field Research**<br><br>Include the following details if this study involves the collection of plant, animal, or other materials from a natural setting:<br>• Field permit number<br>• Name of the institution or relevant body that granted permission	
**Data Availability**<br><br>Authors are required to make all data	Yes - all data are fully available without restriction

Powered by Editorial Manager® and ProduXion Manager® from Aries Systems Corporation

underlying the findings described fully available, without restriction, and from the time of publication. PLOS allows rare exceptions to address legal and ethical concerns. See the PLOS Data Policy and FAQ for detailed information.<br><br>A Data Availability Statement describing where the data can be found is required at submission. Your answers to this question constitute the Data Availability Statement and **will be published in the article**, if accepted.<br><br>**Important:** Stating 'data available on request from the author' is not sufficient. If your data are only available upon request, select 'No' for the first question and explain your exceptional situation in the text box.<br><br>Do the authors confirm that all data underlying the findings described in their manuscript are fully available without restriction?	
**Describe where the data may be found in full sentences. If you are copying our sample text, replace any instances of XXX with the appropriate details.**<br><br>• If the data are **held or will be held in a public repository**, include URLs, accession numbers or DOIs. If this information will only be available after acceptance, indicate this by ticking the box below. For example: *All XXX files are available from the XXX database (accession number(s) XXX, XXX.*).<br>• If the data are all contained **within the manuscript and/or Supporting Information files**, enter the following: *All relevant data are within the manuscript and its Supporting Information files.*<br>• If neither of these applies but you are able to provide **details of access elsewhere**, with or without limitations, please do so. For example:<br><br>*Data cannot be shared publicly because of [XXX]. Data are available from the*	All relevant data are within the manuscript and its Supporting Information files

XXX Institutional Data Access / Ethics Committee (contact via XXX) for researchers who meet the criteria for access to confidential data.<br><br>*The data underlying the results presented in the study are available from (include the name of the third party and contact information or URL).*<br>• This text is appropriate if the data are owned by a third party and authors do not have permission to share the data.<br><br>* typeset	
Additional data availability information:	Tick here if your circumstances are not covered by the questions above and you need the journal's help to make your data available.

Powered by Editorial Manager® and ProduXion Manager® from Aries Systems Corporation

Manuscript Click here to access/download;Manuscript;Manuscript.docx

Full title: Acid-base and metabolic parameters and cerebral oxygenation during the immediate transition after birth - An two-center observational study

Short title: Acid-base status and metabolism and cerebral oxygenation after birth.

Authors: Christian Mattersberger[1], Nariae Baik-Schneditz[1,2], Bernhard Schwaberger[1,2], Georg M. Schmölzer[3,4], Lukas Mileder[1,2], Berndt Urlesberger[1,2], Gerhard Pichler[1,2*]

Affiliation:
[1]Department of Paediatrics and Adolescent Medicine, Division of Neonatology, Medical University of Graz, Graz, Styria, Austria;
[2]Research Unit for Neonatal Micro- and Macrocircultation, Department of Paediatrics and Adolescent Medicine, Medical University of Graz, Graz, Styria, Austria;
[3]Centre for the Studies of Asphyxia and Resuscitation, Royal Alexandra Hospital, Edmonton, Canada;
[4]Department of Paediatrics, University of Alberta, Edmonton, Canada;

* Corresponding author
E-mail: gerhard.pichler@medunigraz.at (GP)

Abstract:

Objective: The association between blood glucose level and cerebral oxygenation (cerebral regional oxygen saturation [$crSO_2$] and cerebral fractional tissue oxygen extraction [FTOE]) in neonates has already been described. Aim of the present study was to investigate if acid-base and other metabolic parameters have an impact on cerebral oxygenation immediately after birth in preterm and term neonates.

Study design: Post-hoc analyses of secondary outcome parameters of two prospective observational studies were performed. Preterm and term neonates born by caesarean section were included, in whom i) cerebral near-infrared spectroscopy (NIRS) measurements were performed during the first 15 minutes after birth and ii) a capillary blood gas analysis was performed between 10 and 20 minutes after birth. Vital signs were routinely monitored with pulse oximetry (arterial oxygen saturation [SpO_2] and heart rate [HR]). Correlation analyses were performed to investigate potential associations between acid-base and metabolic parameters (lactate [LAC], pH-value [pH], base-excess [BE] and bicarbonate [HCO_3]) from capillary blood and NIRS-derived $crSO_2$ and FTOE at 15 minutes after birth.

Results: One-hundred-fifty-seven neonates, 42 preterm neonates (gestational age [IQR] 34.0 weeks [3.3], birth weight [IQR] 1845g [592]) and 115 term neonates (gestational age [IQR] 38.9 weeks [1.0], birth weight [IQR] 3230g [570]) were included in the study.

Median $crSO_2$ [IQR] at 15 minutes after birth were 82% [16] in preterm neonates and 83% [12] in term neonates. Median FTOE [IQR] values at 15 minutes after birth were 0.13 [0.15] in preterm neonates and 0.14 [0.14] in term neonates.

In preterm neonates, higher LAC and lower pH, and BE were associated with lower $crSO_2$ and higher FTOE. In term neonates, higher HCO_3 was associated with higher FTOE.

Conclusion: There were significant associations between several acid-base and metabolic parameters and cerebral oxygenation in preterm neonates, while in term neonates only HCO_3 correlated positively with FTOE.

List of Abbreviations:

BE	base excess
CO_2	carbon dioxide
$crSO_2$	cerebral regional oxygen saturation
FTOE	fractional tissue oxygen extraction
HCO_3	bicarbonate
HR	heart rate
LAC	lactate
NIRS	near-infrared spectroscopy
pH	pH value
SpO_2	arterial oxygen saturation

Introduction

The transition from intra- to extra-uterine life of a neonate is characterized by independence from placental oxygen and nutrient supply. Disturbances of this transition immediately after birth may lead to impaired oxygen and nutrient supply and, thus, to possible irreversible impairments. Due to its high vulnerability, the neonatal brain should be one of the target organs of clinical observation after birth. The (autoregulation) mechanism to maintain cerebral oxygen and nutrients supply, especially during and after perinatal transition, is still not completely understood.(1),(2),(3) Hyper- and hypoxia during this period can possibly lead to cerebral damages like intraventricular haemorrhage, periventricular leukomalacia or retinopathy of prematurity.(4),(5),(6) The currently recommended monitoring for the assessment of neonates after birth includes peripheral arterial oxygen saturation (SpO_2) and heart rate (HR), measured with pulse oximetry and/or electrocardiogram.(7),(8),(9) Unfortunately, the routinely used methods do not assess cerebral oxygen delivery and oxygen consumption and, therefore, neglect potentially crucial information regarding the cerebral oxygenation.(10) Near-infrared spectroscopy (NIRS) is a non-invasive, real-time method which enables the monitoring of cerebral tissue oxygenation and hemodynamics. Reference ranges for neonatal cerebral oxygenation (cerebral regional tissue oxygen saturation [$crSO_2$] and cerebral fractional tissue oxygen extraction [FTOE]) immediately after birth have already been established for different NIRS devices.(11),(12),(13) One two-center prospective observational study demonstrated an association between lower $crSO_2$ and intraventricular haemorrhage in preterm neonates during the immediate transition.(10) Further, intervention guidelines based on cerebral oxygenation monitoring measured with NIRS aiming at reducing the time of hypoxia during after birth have already been developed.(14),(15)

Cerebral oxygenation is influenced by oxygen delivery, based on vascular resistance and cardiac output, and by oxygen consumption. Both, oxygen delivery and oxygen consumption

may be influenced by the acid-base status and the metabolism. A recent study has already demonstrated a negative association between blood glucose and cerebral oxygenation in preterm and term neonates immediately after birth.(16) However, to date no data are available regarding possible associations between acid-base and metabolic parameters and cerebral oxygenation ($crSO_2$, FTOE) in neonates during immediate transition after birth.

Acid-base and metabolic parameters reflect sufficient oxygenation and the counter regulation mechanism in hypoxic conditions of the organism. During immediate neonatal transition after birth, acid-base and metabolic parameters, measured out of umbilical cord blood, have already been used as outcome predictors and as indicators for further intervention in neonates.(17),(18) A high lactate (LAC) level is an unspecific chemical marker e.g. for neonatal hypoxia and may be associated with an increased risk for adverse neurological outcome.(19),(20) Furthermore, deviations from normal acid-base levels (pH-value [pH], base-excess [BE], and bicarbonate [HCO_3]) during immediate postnatal transition are further predictors for poor neurological outcome in preterm and term neonates.(20),(21),(22) However, there are currently no data available about the influence of changes in the acid-base status and the metabolism and the effect on $crSO_2$ and FTOE in neonates during immediate transition after birth. Therefore, the aim of the present study was to identify additional associations between acid-base and metabolic parameters and their relation to cerebral oxygenation in preterm and term neonates. We hypothesized that the acid-base status and the metabolism are associated with $crSO_2$ and FTOE.

Material and Methods

This study analysed secondary outcome parameters of two prospective observational studies carried out at the Royal Alexandra Hospital, Edmonton, Canada, and at the Division of Neonatology, Medical University of Graz, Austria. The studies were conducted between

February 2014 and February 2015 in Edmonton and from October 2015 to September 2018 in Graz.(23)

Inclusion and exclusion criteria

Preterm and term neonates with the decision to conduct full life support and who were born by caesarean section were included. Neonates with congenital malformations (e.g. congenital diaphragmatic hernia) were excluded. Institutional ethical approvals (Canada: Pro00032233; Graz: 27-465 ex 14/15) were obtained. In Graz written parental consent was obtained before birth, while in Edmonton deferred consent was used with written parental consent after delivery.

Study procedure

The neonates were brought to the resuscitation desk and were placed under an overhead heater in a supine position. Preterm neonates <29 week of gestation were covered in a plastic wrap according the neonatal resuscitation guidelines. Resuscitation was performed by dedicated resuscitation teams (neonatologist/experienced resident and nurse), which were not involved in the study, according the neonatal resuscitation guidelines.(24),(25)

Respiratory support was provided using continuous positive airway pressure or positive pressure ventilation using a T-piece device (Neopuff Infant Resuscitator, Fisher & Paykel Healthcare, Auckland, New Zealand). The level of oxygen was titrated according the neonatal rescuscitation guidelines. For SpO_2 and HR monitoring, a pulse oximetry probe (IntelliVue MP30 Monitor, Philips, Amsterdam, The Netherlands) was placed around the right wrist/hand. For measuring $crSO_2$ and FTOE, an INVOS 5100 monitor (Covidien, Minnesota, USA) with a

neonatal sensor was applied on the left fronto-parietal head in each neonate and fixed with a cohesive conforming bandage.

The multi-channel system alpha-trace digital MM (BEST Medical Systems, Vienna, Austria) was used to store all variables for subsequent analyses. Values of the non-invasive monitoring were stored every second. The 15th minute mean or median value of each neonate was used for analyses. As a quality criterion, $crSO_2$ and SpO_2 values were eliminated if $crSO_2$ was higher than SpO_2.(26) FTOE was calculated by the following formula: ([SpO_2-$crSO_2$]/SpO_2).(27)

Acid-base and metabolic parameters (LAC, pH, BE, and HCO_3), measured from capillary blood samples in the period between 10-20 minutes after birth using a blood gas analyser (ABL 800 Flex, Fa. Drott, Wiener Neustadt, Austria), were used for analyses. The capillary blood samples were taken according to the discretion of the attending neonatologist.

Statistical analysis

Data are presented as mean and standard deviation for normally distributed values, and median with interquartile range (IQR) for data with skewed distribution.

Demographic data and measured parameters (SpO_2, HR, $crSO_2$, FTOE, LAC, pH, BE, HCO_3) of preterm and term neonates were compared. Categorical demographic variables were compared with the Chi-square test or Fisher's exact test. Continuous variables were compared using Student's t-test or Mann-Whitney-U test, as appropriate. The associations between $crSO_2$/FTOE and acid-base and metabolic parameters were analyzed using Spearman's rank correlation coefficient or Pearson's correlation, as appropriate. A p-value <0.05 was considered statistically significant. The statistical analyses were performed using IBM SPSS Statistics 26.0.0 (IBM Corporation, Armonk, NY, USA).

Results

Out of 500 eligible neonates, in whom NIRS measurements were performed, we included 42 preterm and 115 term neonates. Most neonates were excluded because of the lack of a blood sample or $crSO_2$ data at the 15th minute after birth (Fig 1). One hundred forty-two mothers of the included neonates received regional anesthesia, nine mothers received general anesthesia, and in 6 mothers anesthesia was not documented for the caesarean section. Indications for a caesarean section were previous caesarean section s (n=38), intrauterine growth restriction (n=10), multiple birth (n=22), breech presentation (n=10), suspected fetal and/or maternal infection (n=1), transverse presentation (n=1), placenta praevia (n=3), pre-eclampsia and eclampsia (n=2), premature rupture of the membrane and premature labor (n=8), others (n=19) and not documented (n=43).

Fig 1 Patient flow diagram. [SpO_2 = arterial oxygen saturation, $crSO_2$ = cerebral regional oxygen saturation; FTOE = fractional tissue oxygen extraction].

Demographic data of the included neonates are presented in Table 1. Besides gestational age and birth weight, there were significant differences in Apgar scores, SpO_2, and rectally measured central temperature between preterm and term neonates, with lower values in preterm neonates. Blood samples were taken at a median postnatal age of 17 minutes (3 IQR) in preterm and of 16 minutes (2 IQR) in term neonates (p=.051).

Table 1. Demographic and clinical data of the study population. Mean value and interquartile range (IQR) at the 15th minute after birth of preterm and term neonates with group comparison.

		Preterm n = 42	IQR	Term n = 115	IQR	Group comparison p-value
Demographics	Gestational age **(weeks)**	34.0	3.3	38.9	1	<.001
	Birth weight **(g)**	1845.0	592	3230.0	570	<.001
	Apgar **1 min**	8	1	9	0	<.001
	Apgar **5 min**	9	2	10	1	<.001
	Apgar **10 min**	10	1	10	0	<.001
Monitoring	Mean arterial blood pressure **(mmHg) ***	43	12	45	11	.065
	Rectal temperature **(C°) ***	36.8	0.5	37	0.3	.002
	Arterial oxygen saturation **(%) ***	95	7	96	4	.004
	Heart rate **(bpm) ***	155	23	152	21	.548
	Cerebral regional oxygen saturation **(%) ***	82	16	83	12	.144
	Fractional tissue oxygen extraction *	0.13	0.15	0.14	0.14	.333
Ventilation	Supplemental oxygen, **n (%)**	12	(29)	9	(8)	<.001
	Non-invasive respiratory support, **n (%)**	19	(45)	19	(17)	<.001
	Endotracheal intubation, **n (%)**	4	(10)	0	(0)	.019
Acid-base metabolism	Lactate **(mg/dL) ***	2.8	1.1	2.7	1.2	.346
	pH *	7.267	0.095	7.293	0.061	<.001
	Base Excess **(mmol/L) ***	- 2.3	2.6	- 0.9	2.8	.001
	Bicarbonate **(mmol/L) ***	21.0	2.6	21.6	1.9	.126
	Time of blood sample, postnatal **(min)**	17	3	16	2	.051

* = 15 minutes postnatal.

During the study period, 12 preterm (28.6%) and nine term (7.8%) neonates received supplemental oxygen (p=<.001). 19 preterm (45.2%) and 19 term (7.8%) neonates received non-invasive respiratory support (p=<.001). Four preterm (9.5%) neonates, but no term neonate, were intubated and mechanically ventilated (p=.019) (Table 1).

Cerebral tissue oxygenation

There was no significant difference in $crSO_2$ and FTOE between preterm and term neonates. (Table 1). Six (14.3%) preterm neonates had $crSO_2$ values <10th centile and nine (21.4%) >90th centile at 15 minutes after birth according to published reference ranges. Four (3.5%) term

neonates had $crSO_2$ values <10th centile and 24 (20.9%) >90th centile. In five (11.9%) preterm neonates FTOE values were <10th centile and in three (7.1%) >90th centile at 15 minutes after birth. Twenty-nine (25.2%) term neonates had FTOE values <10th centile and three (2.6%) >90th centile. (12)

Acid-base and metabolic parameters

There was a significant difference in pH, and BE between preterm and term neonates, with lower values in preterm neonates.

One (0.8%) term neonate had LAC <2.5th centile and 10 (8.5%) >97.5th centile after birth according to the centiles published by Cousineau et al. (28). Seventy-four (63.2%) term neonates had pH values <2.5th centile and no neonate had pH values >97.5th centile at 15 minutes after birth. (28)

Correlation analyses of acid-base and metabolic parameters and cerebral tissue oxygenation

Table 2 shows the correlation analyses of cerebral oxygenation and acid-base and metabolic parameters. Scatter plots of acid-base and metabolic parameters and cerebral oxygenation for preterm and term neonates are provided in Fig 2.

Fig 2 Scatter plots of acid-base and metabolic parameters and cerebral oxygenation (cerebral regional oxygen saturation [$crSO_2$] and fractional-tissue oxygen extraction [FTOE]) in preterm and term neonates.

Table 2. Spearman/Pearson correlation analyses of acid-base and metabolic parameters and cerebral oxygenation in preterm and term neonates 15 minutes after birth.

	Preterm (n = 42)				***Term (n = 115)***			
	crSO2 (%)	p-Value	FTOE	p-Value	crSO2 (%)	p-Value	FTOE	p-Value
Lactate ***(mg/dL)***	ϱ =-.388	.015	ϱ = .379	.019	ϱ =-.128	.185	ϱ = .118	.244
pH	ϱ = .585	<.001	ϱ =-.489	.001	ϱ =-.129	.172	ϱ = .163	.083
Base Excess ***(mmol/L)***	ϱ = .355	.021	ϱ =-.314	.045	ϱ =-.144	.128	ϱ = .179	.058
Bicarbonate ***(mmol/L)***	ϱ = .285	.079	ϱ =-.206	.215	ϱ =-.173	.069	ϱ = .216	.023

ϱ = Person/Spearman correlation coefficient.

In preterm neonates, correlation analyses between $crSO_2$ and LAC (p=-.388, q=.015) showed a significantly negative correlation, while FTOE and LAC (p=.379, q=.019) were positively correlated. In preterm neonates, correlation analyses between $crSO_2$ and pH and BE showed a significant positive correlation and FTOE were negatively correlated with pH and BE.

In term neonates, there were no significant correlations between $crSO_2$ and LAC, pH, BE, HCO_3, but a significantly positive correlation between FTOE and HCO_3.

Discussion

To our knowledge, the present study is the first to demonstrate several significant correlations between acid-base and metabolic parameters and cerebral oxygenation especially in preterm, but also in term neonates during the immediate transition after birth.

Previous investigations on correlations between cerebral oxygenation and acid-base and metabolic parameters during the neonatal period were controversial. Outside the transitional period after birth, some studies demonstrated correlations between cerebral tissue oxygenation and acid-base and metabolic parameters(29),(30),(31),(32),(33),(34) whereas others found none at all(35),(36),(37),(38),(39),(40),(41) in neonates. There are several possible reasons for these different findings, among them the gestational age, the postnatal age at the time of measurement, the numbers of neonates included and, therefore, the statistical power, and the timing of measurements of cerebral oxygenation and acid-base and metabolic parameters.

Lactate and cerebral oxygenation

LAC is a metabolite within the anaerobic energy maintenance system and is increased during inadequate cellular oxygenation.(42) Our study demonstrated a negative correlation between $crSO_2$ and LAC and a positive correlation between FTOE and LAC in preterm, but not in term neonates during the immediate transition after birth. Similar results have been published for extremely preterm neonates during the first days after birth (33). In contrast, other studies of critically-ill neonates and infants who had been free of cerebral disease or trauma for up to one year (35), of paediatric patients during heart surgery (31), and of neonates with prenatally diagnosed congenital heart disease during the first 72 hours after birth (39) did not demonstrate any correlations.

pH and cerebral oxygenation

Both, respiratory as well as metabolic acidosis may result from inadequate oxygen delivery and impaired gas exchange. The pH level of umbilical artery blood is the most sensitive indicator of fetal hypoxia during delivery and is used for evaluating postnatal adaptation.(43) We found a positive correlation between $crSO_2$ and pH measured from a capillary blood sample 15min after birth and a negative correlation between FTOE and pH in preterm, but not in term neonates during the immediate transition after birth. There was only a trend towards a positive correlation between FTOE and pH measured from a capillary blood sample in term neonates. Similar results of a positive correlation between pH and $crSO_2$ have been published for neonates shortly before birth (29) and in neonates and infants undergoing thoracoscopic repair of congenital diaphragmatic hernia and esophageal atresia (30). In contrast, a negative correlation between $crSO_2$ and pH was demonstrated in paediatric patients undergoing surgical heart procedures (31) and in term-born infants suffering from hypertrophic pyloric stenosis during correction of metabolic alkalosis (32). Against them, some studies demonstrated the absence of correlations between $crSO_2$ or FTOE and pH in i) critically-ill neonates and infants who have been free of cerebral disease and trauma for up to 1 year (35), ii) in very preterm neonates during the first 6 hours of life (36), iii) in neonates and children during cardiopulmonary bypass surgery (37), iv) in extremely preterm neonates with very low birth weight during sodium HCO_3 infusion for correction of metabolic acidosis during the first postnatal week (38), v) in term born infants with prenatally diagnosed congenital heart disease during the first 72 hours after birth (39), vi) in clinically stable preterm infants in the intensive care unit (40) and vii) in term neonates with persisting pulmonary hypertension (41).

Base excess and cerebral oxygenation

The BE quantifies the magnitude of the metabolic acidosis and represents a risk factor for central neurologic injury and is a prognostic marker for short- and long-term outcome in asphyxiated neonates.(44),(45) In our study, BE was positively correlated with $crSO_2$ and negatively with FTOE in preterm neonates during the immediate transition after birth. Again, there was only a trend towards a positive correlation between BE and FTOE in term neonates. Studies demonstrated a negative correlation between the cerebral oxygenation and BE i)in fetuses shortly before birth (29), ii) in term-born infants suffering from hypertrophic pyloric stenosis (32) and iii) in extremely preterm neonates receiving sodium HCO_3 during the first 24 hours after birth (34). In contrast, other studies found no correlation between BE and $crSO_2$ or FTOE i) in critically-ill neonates and infants up to one year (35), ii) in extremely preterm neonates with very low birth weight during sodium HCO_3 therapy due to metabolic acidosis during the first week of life (38) and iii) in clinically stable preterm infants in the neonatal intensive care unit (40).

Bicarbonate and cerebral oxygenation

Buffers are important for proton elimination and, particularly, HCO_3 is the most important buffer system and contributes approximately 35% to this effect.(46) Our study showed a positive correlation between $crSO_2$ and HCO_3 and a negative correlation between FTOE and HCO_3 in preterm. Further, FTOE correlated positively with HCO_3 and $crSO_2$ showed a trend towards a negative correlation to HCO_3 in term neonates during the first 15 minutes after birth. Similar results have been published in extremely preterm neonates during sodium HCO_3 administration during the first 24 postnatal hours (34). In contrast, another study reported a negative correlation between cerebral oxygenation and HCO_3 in term-born infants with hypertrophic pyloric stenosis suffering from metabolic alkalosis (32). The absence of a correlation between $crSO_2$ and HCO_3 has been described in extremely preterm neonates with

very low birth weight and metabolic acidosis during the first week after birth (38) and in clinically stable preterm infants receiving treatment in the neonatal intensive care unit (40).

There may be different explanations for our results, which are in part contradictory to other findings.

A possible explanation of the observed negative correlation between $crSO_2$ and LAC is that increase in LAC leads to a pulmonary vasoconstriction and a reduction in cardiac stroke volume as a result of reduced cardiac contractility.(47) Therefore, increase in LAC levels might lead to impaired cardiac output and thus to a decrease of oxygen delivery to the brain, followed by a reduced $crSO_2$. Furthermore, animal studies demonstrated that with increase in LAC the pH level decreases with potential effects on the neonatal heart.(47),(48) This effect of LAC on the neonatal heart seems to concern only preterm neonates. A recent study did not demonstrate this effect of LAC on the cardiac output in term born neonates during the first 6 hours after birth.(49)

Concerning pH, we observed a positive correlation between $crSO_2$ and pH in preterm neonates but not in term neonates. In vitro investigations demonstrated that acidosis leads to a reduced contractility of cardiomyocytes, and to a reduced responsiveness on catecholamines.(50) Both observations may subsequently result in a reduced cardiac output, and thus, may lead to a decrease in oxygen delivery to the brain with reduced $crSO_2$. Though, in contrast to these laboratory findings, the myocardial contractility of hemodynamically stable preterm neonates during the transitional period remains relatively unaffected during acidosis, even at pH values below 7.00.(51) There seems to be no relationship between pH and cardiac output in preterm neonates during the first 3 days after birth.(51)

In addition, depending on the tissue, severe acidosis or alkalosis leads to a vasodilatation or vasoconstriction. On the one side, acidosis leads to an increased pulmonary vascular resistance

and raise in pulmonary artery pressure. On the other side, acidosis leads to a redistribution of blood from peripheral veins into the lungs with consequential increase in preload and following the frank starling mechanism an increase in cardiac output.(52) In preterm neonates after the transitional period defined as days 4-14 after birth, acidosis leads to a decrease in systemic vascular resistance with subsequent increase in left ventricular output. In preterm neonates during the transitional period defined as days 1-3, there seems to be no relationship between the pH and the systemic vascular resistance. Following this, the systemic vascular response to acidosis undergoes a postnatal maturational process in preterm neonates during the first two postnatal weeks.(51)

Beside the vasodilative effect of acidosis on the systemic vascular resistance, acidosis leads to vasodilation of cerebral vessels resulting in an increase in cerebral blood flow and oxygenation.(31) Therefore one would expect a negative correlation of pH and crSO2. However, we observed a positive correlation of pH and crSO2. An immature compensation mechanism with inadequate vasodilation due to acidosis in preterm neonates might explain our finding.

A possible explanations of our observed positive correlation between BE and $crSO_2$ in preterm neonates are the also observed correlations of crSO2 with other acid-base parameters since BE is calculated out of acid-base parameters.(55) However, BE is an indicator of shock and efficacy of resuscitation and reflective of volume deficit.(56) It can be assumed that decreased BE demonstrates a centralisation with its resulting hemodynamic problems for the oxygen delivery that is in accordance with the present findings in preterm neonates.

Concerning HCO_3 we observed a trend towards a positive correlation with crSO2 and a trend towards negative correlation with FTOE in preterm neonates. This observation is in accordance with the findings in pH and crSO2 and FTOE in preterm neonates, since pH and HCO3 are strongly linked together.

In term neonates there was a positive significant correlation of HCO3 with FTOE and a trend towards a negative correlation to crSO2, which again is in accordance with the findings in pH and crSO2 and FTOE in term neonates.

Based on the observed multiple significant correlations between acid-base status and cerebral oxygenation especially in preterm neonates in comparison to term neonates, we hypothesize that the capacity of the cerebral autoregulation mechanism to maintain constant cerebral oxygen supply is less dependent on acid-base status with increasing gestational age. Given this assumption, an increase in gestational age obviously leads to an increased ability to compensate an impaired cerebral oxygenation in neonates during the immediate neonatal transition after birth.

One of the strengths of this investigation is that it was a two center study with a rather high sample size especially regarding term neonates. Limitations include the smaller sample size of preterm neonates. Nonetheless, we still found significant associations in preterm neonates. A further limitation is the difference in timing between the NIRS measurements and the taking of blood samples. Nevertheless, it can be assumed that the components of the acid-base metabolism did not change significantly between NIRS measurements until minute 15 after birth and blood sample taking. Another limitation of our study are possible interactions of the investigated acid-base and metabolic parameters, which may have potentially confounded the observed effects on cerebral oxygenation. Finally, blood gas analysis use the Van Slyke equation to calculate various acid-base variables by measuring the pH, hemoglobin content and partial pressure of CO_2.(62) This could be a further confounder for the observed correlation.

Conclusion

Fifteen minutes after birth, acid-base and metabolic parameters are significantly associated with $crSO_2$ and FTOE in preterm neonates. An increase of LAC, and a decrease of pH and BE,

all three are associated with a decrease in $crSO_2$ and an increase in FTOE in preterm neonates only. In term neonates. there is a significant positive correlation between HCO_3 and FTOE. Still, the causal relationship between acid-base and metabolic parameters and cerebral oxygenation during the immediate postnatal transition, and thus potential influences on short- and long-term neonatal outcomes, have to be evaluated.

References:

1. Riera J, Hyttel-Sorensen S, Bravo MC, Cabañas F, López-Ortego P, Sanchez L, et al. The SafeBoosC phase II clinical trial: An analysis of the interventions related with the oximeter readings. Arch Dis Child Fetal Neonatal Ed. 2016;101(4):F333–8.
2. Tzeng Y-C, Ainslie PN. Blood pressure regulation IX: cerebral autoregulation under blood pressure challenges. Eur J Appl Physiol 2014 Mar 5;114(3):545–59.
3. Greisen G. Autoregulation of cerebral blood flow in newborn babies. Early Hum Dev 2005 May;81(5):423–8.
4. Davis PG, Tan A, O'Donnell CPF, Schulze A, Niermeyer S, Kattwinkel, et al. Resuscitation of newborn infants with 100% oxygen or air: a systematic review and meta-analysis. Lancet (London, England). 2000;364(9442):1329–33.
5. Plomgaard AM, Alderliesten T, Austin T, Van Bel F, Benders M, Claris O, et al. Early biomarkers of brain injury and cerebral hypo- and hyperoxia in the SafeBoosC II trial. PLoS One. 2017 Mar 1;12(3).
6. Yiş U, Kurul SH, Kumral A, Cilaker S, Tuğyan K, Genç Ş, et al. Hyperoxic exposure leads to cell death in the developing brain. Brain Dev. 2008 Oct;30(9):556–62.
7. O'Donnell CPF, Kamlin COF, Davis PG, Morley CJ. Feasibility of and delay in obtaining pulse oximetry during neonatal resuscitation. J Pediatr. 2005;147(5):698–9.
8. Finer N, Leone T. Oxygen saturation monitoring for the preterm infant: the evidence basis for current practice. Pediatr Res. 2009 Apr;65(4):375–80.
9. Dawson JA, Morley CJ. Monitoring oxygen saturation and heart rate in the early neonatal period. Semin Fetal Neonatal Med. 2010 Aug;15(4):203–7.
10. Baik N, Urlesberger B, Schwaberger B, Schmolzer GM, Avian A, Pichler G. Cerebral haemorrhage in preterm neonates: does cerebral regional oxygen saturation during the immediate transition matter? Arch Dis Child Fetal Neonatal Ed. 2015;100(5):F422-7.
11. Urlesberger B, Grossauer K, Pocivalnik M, Avian A, Müller W, Pichler G. Regional oxygen saturation of the brain and peripheral tissue during birth transition of term infants. J Pediatr. 2010;157(5):740–4.
12. Pichler G, Binder C, Avian A, Beckenbach E, Schmölzer GM, Urlesberger B. Reference Ranges for Regional Cerebral Tissue Oxygen Saturation and Fractional Oxygen Extraction in Neonates during Immediate Transition after Birth. J Pediatr. 2013 Dec;163(6):1558–63.
13. Baik N, Urlesberger B, Schwaberger B, Schmölzer GM, Mileder L, Avian A, et al. Reference Ranges for Cerebral Tissue Oxygen Saturation Index in Term Neonates during Immediate Neonatal Transition after Birth. Neonatology. 2015;108:283–6.
14. Pichler G, Urlesberger B, Baik N, Schwaberger B, Binder-Heschl C, Avian A, et al. Cerebral Oxygen Saturation to Guide Oxygen Delivery in Preterm Neonates for the Immediate Transition after Birth: A 2-Center Randomized Controlled Pilot Feasibility Trial. J Pediatr. 2016 Mar;170:73-78.e4.

15. Hyttel-Sorensen S, Austin T, van Bel F, Benders M, Claris O, Dempsey E, et al. A phase II randomized clinical trial on cerebral near-infrared spectroscopy plus a treatment guideline versus treatment as usual for extremely preterm infants during the first three days of life (SafeBoosC): study protocol for a randomized controlled trial. Trials. 2013;14.

16. Mattersberger C, Baik-Schneditz N, Schwaberger B, Schmölzer GM, Mileder L, Pichler-Stachl E, et al. Blood Glucose and Cerebral Tissue Oxygenation Immediately after Birth-An Observational Study. J Pediatr. 2018 Sep 1;200:19–23.

17. Blickstein I, Green T. Umbilical Cord Blood Gases. Clin Perinatol. 2007 Sep;34(3):451-9.

18. Hankins GD V, Speer M. Defining the Pathogenesis and Pathophysiology of Neonatal Encephalopathy and Cerebral Palsy. Obstet Gynecol. 2003 Sep;102(3):628-36.

19. Piquard F, Schaefer A, Dellenbach P, Mkdico-chirurgical C. Is fetal acidosis in the human fetus maternogenic during labor ? A reanalysis. Am J Physiol. 1991 Nov;261(5 Pt 2):R1294-9.

20. Shah S, Tracy M, Smyth J. Postnatal lactate as an early predictor of short-term outcome after intrapartum asphyxia. Vol. 24, Journal of Perinatology. Nature Publishing Group; 2004. p. 16–20.

21. Low JA, Panagiotopoulos C, Derrick EJ. Newborn complications after intrapartum asphyxia with metabolic acidosis in the preterm fetus. Am J Obstet Gynecol. 1995;172(3):805–10.

22. Lavrijsen SW, Uiterwaal CSPM, Stigter RH, de Vries LS, Visser GHA, Groenendaal F. Severe Umbilical Cord Acidemia and Neurological Outcome in Preterm and Full-Term Neonates. Neonatology. 2005 Jul;88(1):27–34.

23. Bresesti I, Bruckner M, Mattersberger C, Baik-Schneditz N, Schwaberger B, Mileder L, et al. Feasibilty of Transcutaneous pCO2 Monitoring During Immediate Transition After Birth—A Prospective Observational Study. Front Pediatr. 2020 Jan 29;8:11.

24. Wyckoff MH, Aziz K, Escobedo MB, Kapadia VS, Kattwinkel J, Perlman JM, et al. Part 13: Neonatal Resuscitation. Circulation. 2015 Oct 20;132(18):S543–60.

25. Wyllie J, Bruinenberg J, Roehr CC, Rüdiger M, Trevisanuto D, Urlesberger B. European Resuscitation Council Guidelines for Resuscitation 2015. Section 7. Resuscitation and support of transition of babies at birth. Resuscitation. 2015;95:249–63.

26. Pocivalnik M, Pichler G, Zotter H, Tax N, Müller W, Urlesberger B. Regional tissue oxygen saturation: comparability and reproducibility of different devices. J Biomed Opt. 2011;16(5):057004.

27. Naulaers G, Meyns B, Miserez M, Leunens V, Van Huffel S, Casaer P, et al. Use of tissue oxygenation index and fractional tissue oxygen extraction as non-invasive parameters for cerebral oxygenation: A validation study in piglets. Neonatology. 2007;92(2):120–6.

28. Cousineau J, Anctil S, Carceller A, Gonthier M, Delvin EE. Neonate capillary blood gas reference values. 2005;38:905–7.

29. Aldrich C.J.; D'Antona D.; Wyatt J.S.; Spencer J.A.D.; Peebles D.M.; Reynolds E.O.R. Fetal cerebral oxygenation measured by near-infrared spectroscopy shortly before birth and acid-base status at birth. Obs Gynecol 1994 84/5. 1994;50:116–7.

30. Bishay M, Giacomello L, Retrosi G, Thyoka M, Nah SA, Mchoney M, et al. Decreased cerebral oxygen saturation during thoracoscopic repair of congenital diaphragmatic hernia and esophageal atresia in infants. J Pediatr Surg. 2011;46(1):47–51.

31. Amigoni A, Mozzo E, Brugnaro L, Tiberio I, Pittarello D, Stellin G, et al. Four-side near-infrared spectroscopy measured in a paediatric population during surgery for congenital heart disease. Interact Cardiovasc Thorac Surg. 2011;12(5):707–12.

32. Nissen M, Cernaianu G, Thränhardt R, Vahdad MR, Barenberg K, Tröbs R-B. Does metabolic alkalosis influence cerebral oxygenation in infantile hypertrophic pyloric stenosis. J Surg reserach. 2017 May 15;212:229-237.

33. Janaillac M, Beausoleil TP, Barrington KJ, Raboisson MJ, Karam O, Dehaes M, et al. Correlations between near-infrared spectroscopy, perfusion index, and cardiac outputs in extremely preterm infants in the first 72 h of life. Eur J Pediatr. 2018;177(4):541–50.

34. Katheria AC, Brown MK, Hassan K, Poeltler DM, Patel DA, Brown VK, et al. Hemodynamic effects of sodium bicarbonate administration. J Perinatol. 2017 May 1;37(5):518–20.

35. Weiss M, Dullenkopf A, Kolarova A, Schulz G, Frey B, Baenziger O. Near-infrared spectroscopic cerebral oxygenation reading in neonates and infants is associated with central venous oxygen saturation. Paediatr Anaesth. 2005;15(2):102–9.

36. von Siebenthal K, Keel M, Fauchère J-C, Dietz V, Haensse D, Wolf U, et al. Variability of Cerebral Hemoglobin Concentration in Very Preterm Infants During the First 6 Hours of Life. Adv Exp Med Biol. 2005;566:91-7.2005;91–7.

37. Menke J, Möller G. Cerebral near-infrared spectroscopy correlates to vital parameters during cardiopulmonary bypass surgery in children. Pediatr Cardiol. 2013;35(1):155–63.

38. Mintzer JP, Parvez B, Alpan G, LaGamma EF. Effects of sodium bicarbonate correction of metabolic acidosis on regional tissue oxygenation in very low birth weight neonates. J Perinatol. 2015;35(8):601–6.

39. Mebius MJ, van der Laan ME, Verhagen EA, Roofthooft MT, Bos AF, Kooi EM. Cerebral oxygen saturation during the first 72 h after birth in infants diagnosed prenatally with congenital heart disease. Early Hum Dev. 2016;103:199–203.

40. Hunter CL, Oei JL, Lui K, Schindler T. Cerebral oxygenation as measured by Near-Infrared Spectroscopy in neonatal intensive care: correlation with arterial oxygenation. Acta Paediatr 2017 Jul;106(7)1073-1078

41. Mebius M, Verhagen E, Laan M van der, Bos A. Cerebral oxygen saturation and

extraction in noenates with persistent pulmonary hypertension during the first 72 hours of life. BMJ. 2018;5

42. Armstrong L, Stenson BJ. Use of umbilical cord blood gas analysis in the assessment of the newborn. :430–4

43. Huch A, Huch R, Rooth G. Guidelines for blood sampling and measurement of pH and blood gas values in obstetrics. Eur J Obstet Gynecol Reprod Biol. 1994 May 18;54(3):165–75.

44. Ross MG, Gala R. Use of umbilical artery base excess: Algorithm for the timing of hypoxic injury. Am J Obstet Gynecol. 2002;187(1):1–9.

45. Low JA, Galbraith RS, Raymond MJ, Derrick EJ. Cerebral blood flow velocity in term newborns following intrapartum fetal asphyxia. Acta Paediatr Int J Paediatr. 1994 Oct;83(10):1012–6.

46. Blechner JN. Maternal-fetal acid-base physiology. Vol. 36, Clinical Obstetrics and Gynecology. 1993. p. 3–12.

47. Teplinsky K, O'Toole M, Olman M, Walley KR, Wood LDH. Effect of lactic acidosis on canine hemodynamics and left ventricular function. Am J Physiol - Hear Circ Physiol. 1990;258(4 27-4).

48. Arieff AI, Graf H. Pathophysiology of type A hypoxic lactic acidosis in dogs. Am J Physiol - Endocrinol Metab. 1987;253(3).

49. Eriksen VR, Trautner S, Hahn GH, Greisen G. Lactate acidosis and cardiac output during initial therapeutic cooling in asphyxiated newborn infants. PLoS One. 2019;14(3):1–12.

50. Marsh JD, Margolis TI, Kim D. Mechanism of diminished contractile response to catecholamines during acidosis. Am J Physiol - Hear Circ Physiol. 1988;254(1).

51. Noori S, Wu TW, Seri I. PH effects on cardiac function and systemic vascular resistance in preterm infants. J Pediatr. 2013;162(5):958-963.e1.

52. Mitchell JH, Wildenthal K, Johnson RL. The effects of acid-base disturbances on cardiovascular and pulmonary function. Kidney Int. 1972 May;1(5):375-89.

53. Gortner L, Tutdibi E. Respiratory disorders in preterm and term neonates an update on diagnostics and therapy. Z Geburtshilfe Neonatol. 2011;215(04):145–51.

54. Younus J, Hayat S, Haroon F, Waheed KAI, Khan MQ, Khalid MU. Correlation Of Severity Of Metabolic Acidosis At Admission And Outcome In Asphyxiated Neonates. J Ayub Med Coll Abbottabad. 2020 Apr 1;32(2):189–93.

55. Wiberg N, Källén K, Olofsson P. Base deficit estimation in umbilical cord blood is influenced by gestational age, choice of fetal fluid compartment, and algorithm for calculation. Am J Obstet Gynecol. 2006 Dec 1;195(6):1651–6.

56. Davis JW, Shackford SR, Holbrook TL. Base deficit as a sensitive indicator of compensated shock and tissue oxygen utilization. Surg Gynecol Obstet. 1991;173(6):473–6.

57. Ostrea EM, Odell GB. The influence of bicarbonate administration on blood pH in a "closed system": Clinical implications. J Pediatr. 1972;80:671–80.

58. Loomba RS, Abdulkarim M, Bronicki RA, Villarreal EG, Flores S. Impact of sodium bicarbonate therapy on hemodynamic parameters in infants: a meta-analysis. J Matern Neonatal Med. 2020;0(0):1–7.

59. Fanconi S, Burger R, Ghelfi D, Uehlinger J, Arbenz U. Hemodynamic effects of sodium bicarbonate in critically ill neonates. Intensive Care Med. 1993 Feb;19(2):65–9.

60. Kimmoun A, Novy E, Auchet T, Ducrocq N, Levy B. Hemodynamic consequences of severe lactic acidosis in shock states: From bench to bedside. Crit Care. 2015;19(1).

61. Dix LML, Weeke LC, de Vries LS, Groenendaal F, Baerts W, van Bel F, et al. Carbon Dioxide Fluctuations Are Associated with Changes in Cerebral Oxygenation and Electrical Activity in Infants Born Preterm. J Pediatr. 2017;187:66-72.e1.

62. Siggaard-Andersen O. The van slyke equation. Scand J Clin Lab Invest. 1977;37(S146):15–20.

Click here to access/download;Figure;Fig.1.pdf

Figure 1.

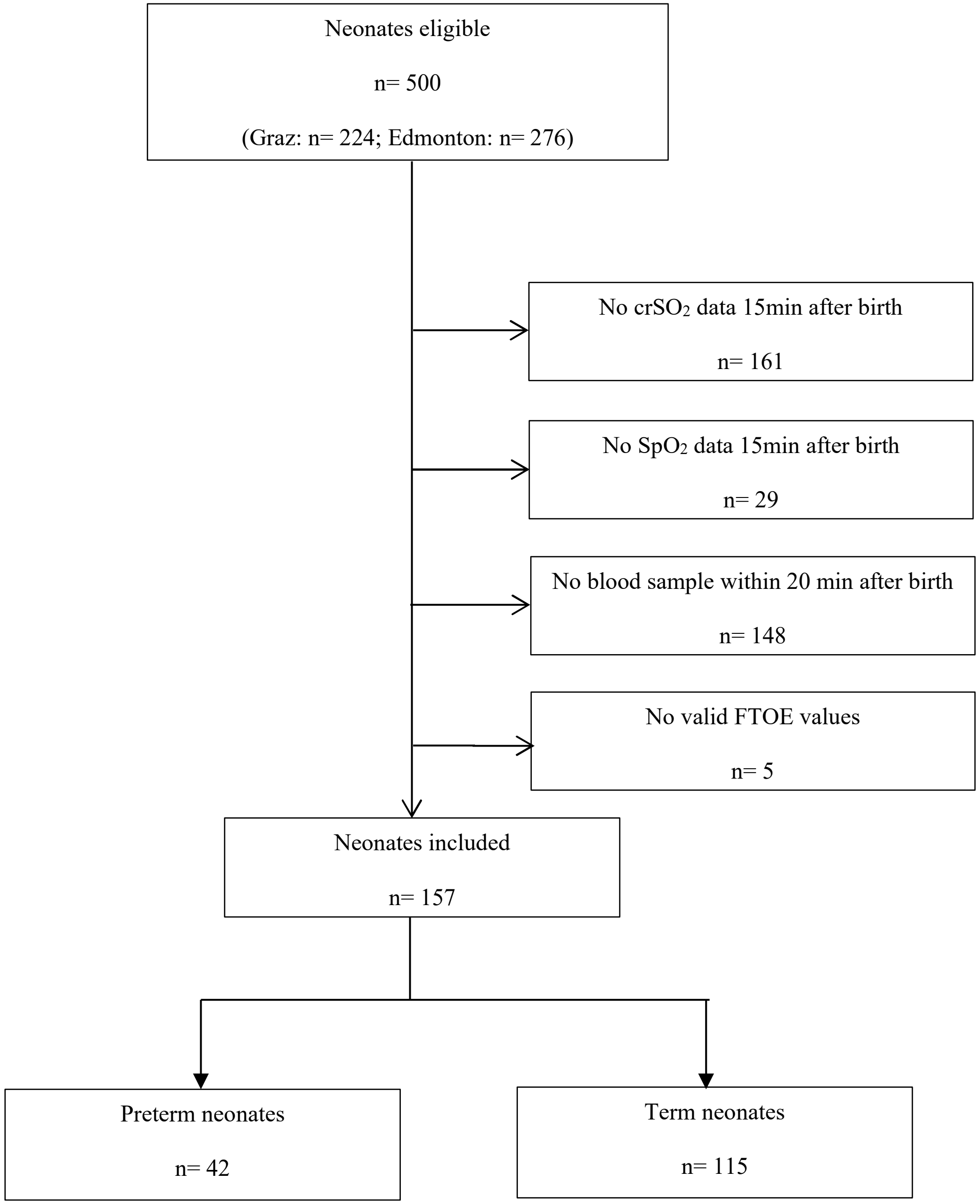

Click here to access/download;Figure;Fig. 2.pdf

Figure 2.

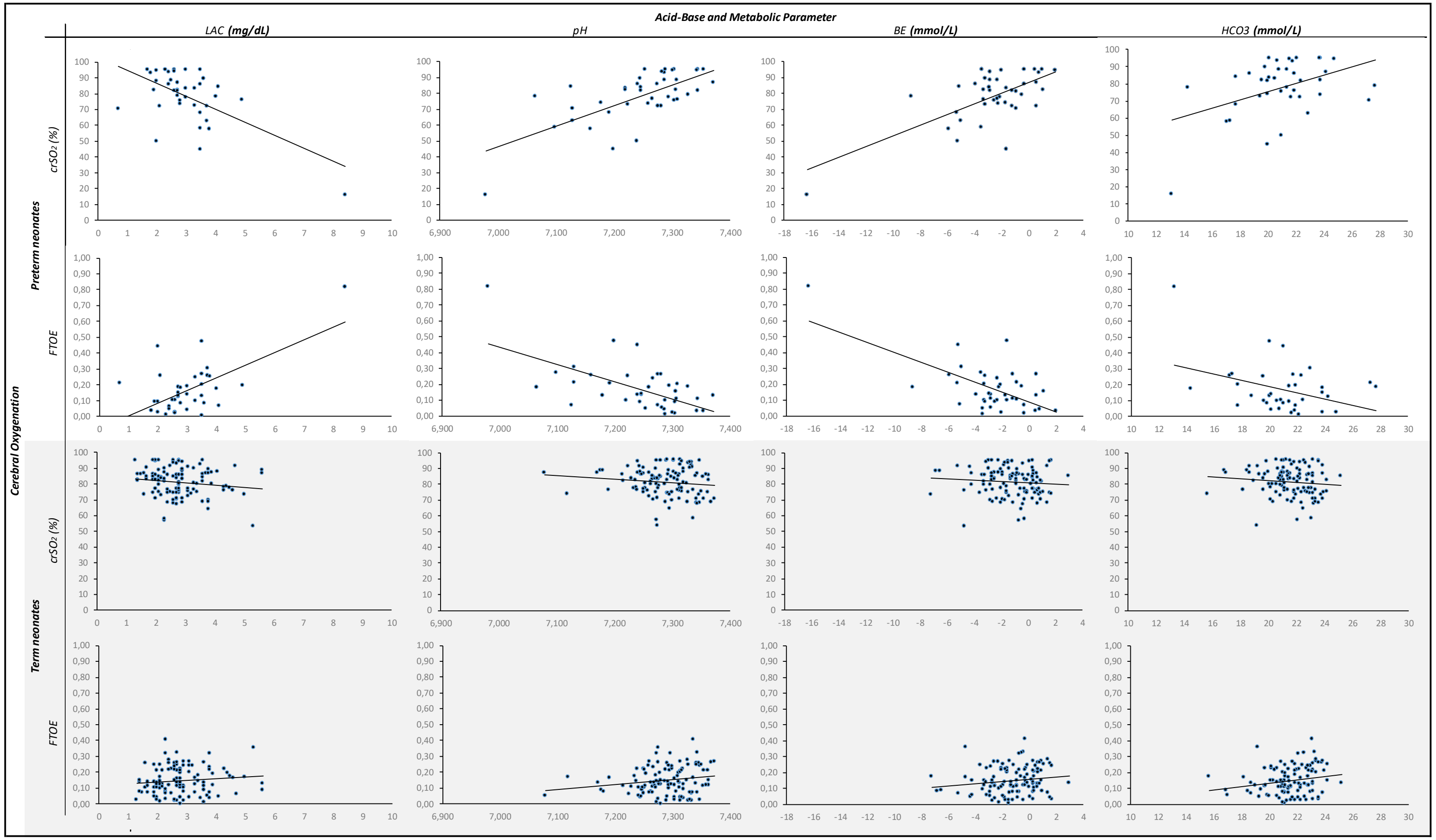

Click here to access/download
Supporting Information
Database.xlsx

Click here to access/download
Supporting Information
REB_approval 1.pdf

Click here to access/download
Supporting Information
REB_approval.pdf
